# Spinocerebellar ataxia type 40: A case report and literature review

**DOI:** 10.1515/tnsci-2020-0190

**Published:** 2021-10-18

**Authors:** Fengyue Han, Dan Su, Chuanqiang Qu

**Affiliations:** Department of Neurology, Shandong Provincial Hospital Affiliated to Shandong First Medical University, Shandong Provincial Hospital, Cheeloo College of Medicine, Shandong University, Jinan, Shandong, 250100, China; Department of Neurology, Jinan Shizhong District People’s Hospital, Jinan, Shandong, 250100, China

**Keywords:** spinocerebellar ataxia, polyglutamine diseases, genotype–phenotype correlations

## Abstract

Spinocerebellar ataxias (SCAs) are a group of neurodegenerative diseases with ataxia as the main clinical manifestation. The phenotypes, gene mutations, and involved sites of different subtypes show a high degree of heterogeneity. The incidence of SCA varies greatly among different subtypes and the case of SCA40 is extremely rare. The aim of this study is to report a rare case of SCA40 and systematically review the incidence, gene mutation, and phenotype of SCAs, especially SCA40.

## 1 Introduction

Autosomal dominant cerebellar ataxias (ADCAs), including spinocerebellar ataxias (SCAs) and episodic ataxias (EAs), are a group of neurodegenerative diseases with ataxia as the main clinical manifestation [1,2]. SCAs present a hereditary and progressive course of the disease, while EA presents paroxysmal and intermittent ataxias. SCAs account for the majority of ADCA, and ADCA usually refers to SCAs [3].

According to different pathogenic genes, SCAs can be divided into different subtypes. With the maturity of DNA sequencing technology and polymerase chain reaction (PCR) technology, more than 40 different subtypes of SCA have been discovered [5]. The incidence of SCAs is about 3/100,000. SCA3 is the most common subtype of SCA worldwide [3,6], followed by SCA1, SCA2, SCA7, and SCA17. These subtypes account for more than 60% of SCAs [7]. The incidence rate of different subtypes varies greatly in different regions due to founder effects [8]. In different races, the prevalence of different subtypes is also different. Through a survey of 14 provinces in Cuba, Velázquez Pérez et al. [9,10] concluded that the prevalence of SCA2 in Holguin was 40.18 per 100,000 inhabitants, which was much higher than 13–18% [11] in the world. And, dentatorubral-pallidoluysian atrophy (DRPLA) is more researched in Japan, and SCA10 has been reported in Mexico [12]. The incidence of other subtypes is very low, which can be considered as rare.

The main clinical manifestation of SCAs is cerebellar ataxia, often accompanied by non-ataxia signs. Rossi et al. analyzed 3,945 patients and found that ataxia is the most common symptom, followed by dysarthria [13]. In addition, the common non-ataxia signs include Parkinson’s syndrome, pyramidal signs retinitis pigmentosa, cognitive dysfunction, epilepsy, peripheral neuropathy, and so on [14]. The phenotypes of different subtypes of SCA have great overlap, especially in the later stage of the disease. So, it is difficult to distinguish different subtypes only by clinical manifestations. However, the presence of certain non-ataxia signs may be instructive for the diagnosis. For example, retinopathy may indicate SCA7 [3], seizures may indicate SCA10, mental retardation may indicate SCA13, and myoclonic epilepsy and dementia may indicate DRPLA [15].

In 2014, Tsoi first reported type 40 SCA caused by coiled-coil domain containing 88c (CCDC88C) gene mutation [16]. This gene is located in 14q32.11-q32.12, and this locus is named SCA40 by the human genome organization (Hugo). CCDC88C is highly expressed in the cerebellum, so the first symptom is usually cerebellar ataxia. Imaging examination usually shows varying degrees of cerebellar atrophy. Cases of SCA 40 are extremely rare. Only Leńska-Mieciek et al. [17] reported one SCA40 family. This study reports a very rare case of SCA40. The incidence, gene mutation, and phenotype of SCA40 were analyzed based on published literatures.

## 2 Case presentation

**Figure 1:**
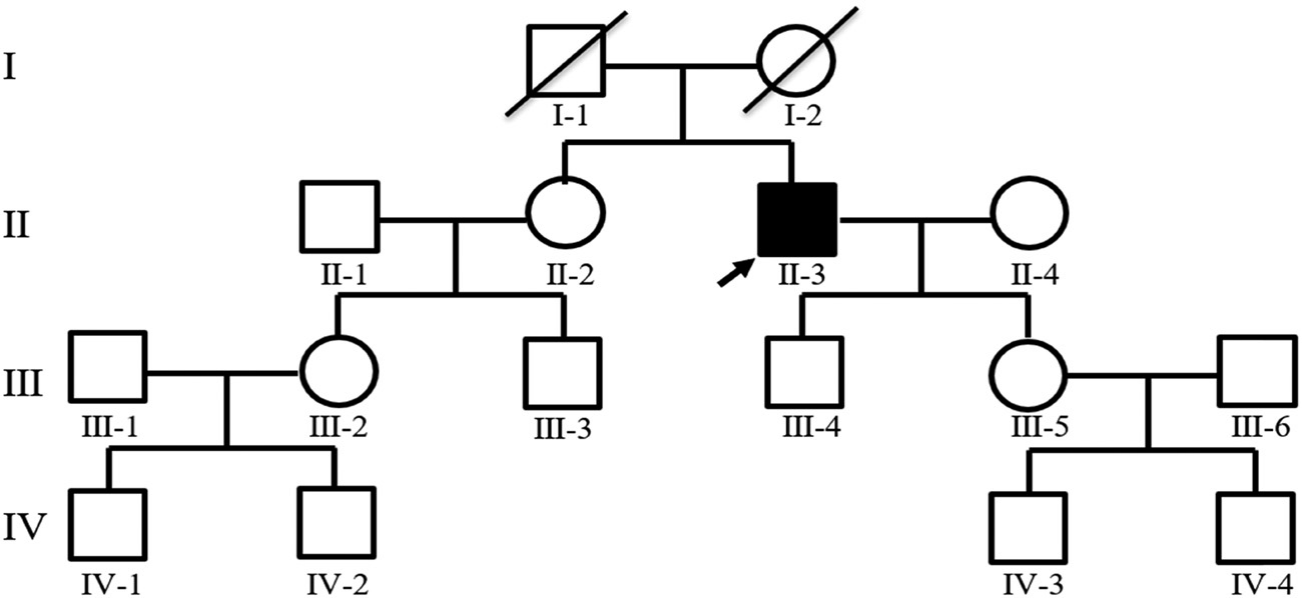
Pedigree of the family with SCA40. Squares indicate males; circles indicate females; an arrow indicates the propositus. Slash marks indicate subjects who are deceased. Roman numerals indicate generations, and Arabic numbers indicate subjects. SCA40, spinocerebellar ataxia type 40.

**Figure 2:**
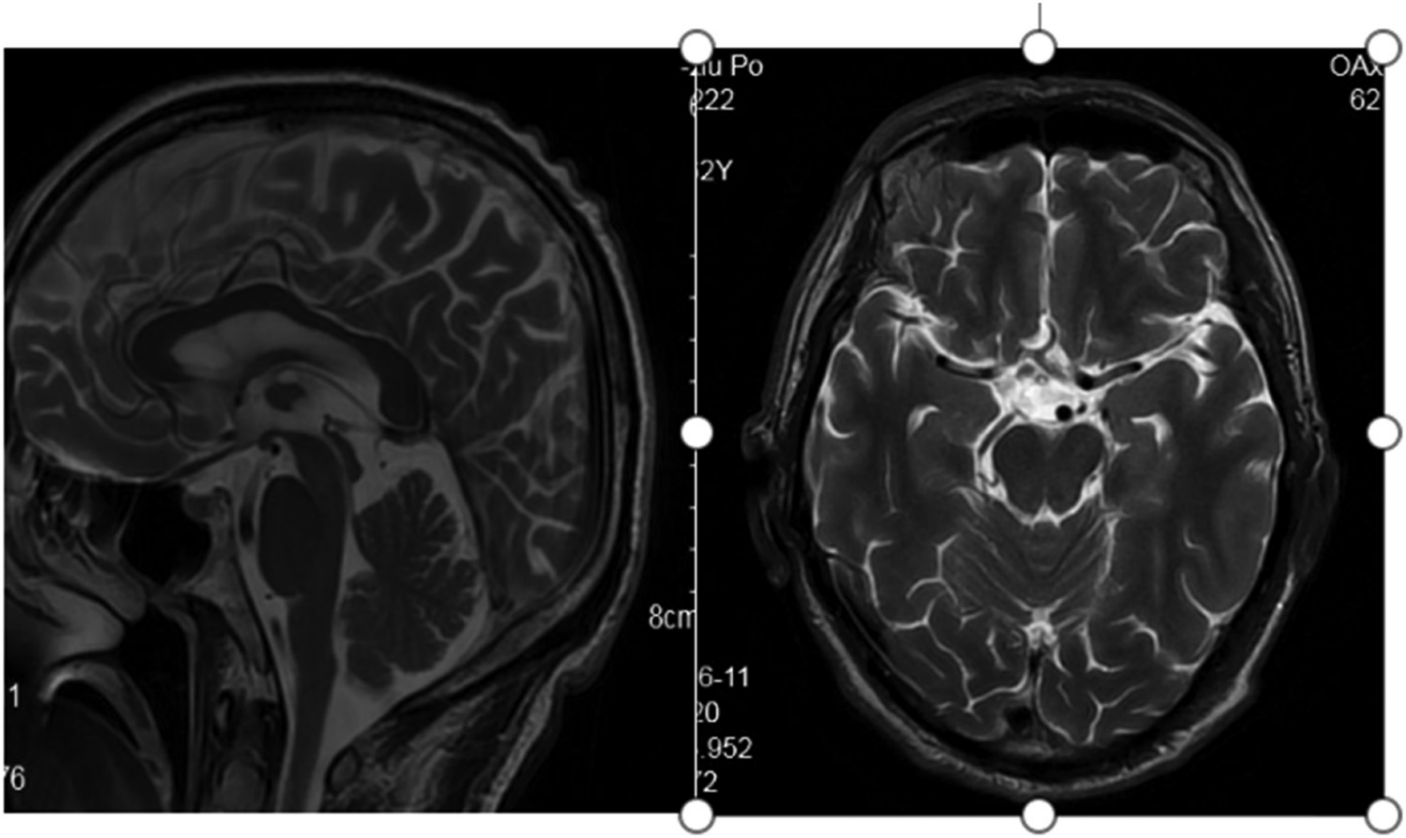
Flat pons, not full and mild cerebellar atrophy in T2-weighted MRI.

**Figure 3:**
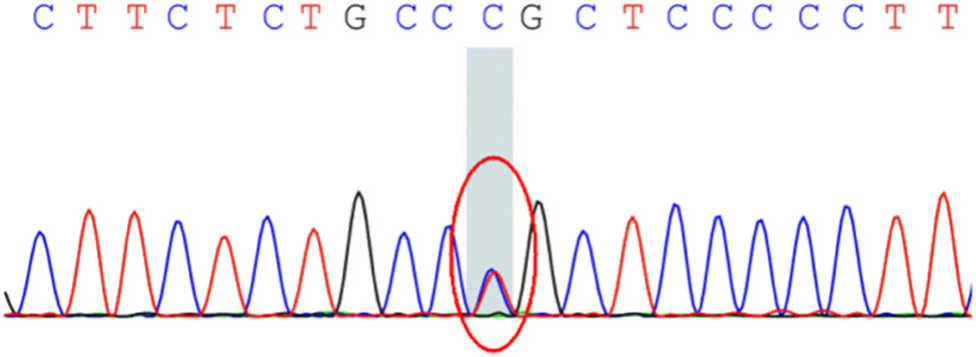
CCDC88C gene mutation sequencing results of II-2, II-3, III-4, III-5, IV-1, and IV-2.


The pedigree of this research is shown in Figure 1. The proband (II-3) is a 61-year-old male who suffered from hypothyroidism and cervical spondylopathy. Two years ago, he experienced shaking of his right hand in a static state, and the shaking was aggravated when he was nervous, accompanied by unresponsiveness. One year ago, he developed speech and swallowing disturbances, and occasionally choking on drinking water. Physical examination revealed involuntary shaking of the right upper limb and clumsy fast alternating hand movements. MRI showed mild cerebellar and pons atrophy (Figure 2). The Mini-Mental State Examination (MMSE) score is 20/30 points (with high school degree). The Hamilton Anxiety Scale (HAMA) score was 16 points, affirming the state of anxiety. A score of 21 on the Hamilton Depression Scale (HAMD) confirmed depression. The Assessment and Rating of Ataxia (SARA) score was 18/40 (Table 1). Inventory of Non-Ataxia Signs (INAs) scored 7 points (Table 2), among which hyperreflexia, spasticity, rigidity, chorea/dyskinesia, dystonia, resting tremor, and cognitive dysfunction were positive. This suggests that the non-ataxia symptoms of the proband are also obvious. Genetic testing of the proband showed a missense mutation in the CCDC88C gene located at 14q32.11 (Figure 3). This mutation is c.1886G > A, so the encoded amino acid changes from arginine to glutamine. The proband’s parents were dead. After detailed inquiry, the parents did not show similar clinical manifestations. The genetic tests revealed heterozygous mutations in the gene CCDC88C in five of the proband’s families (Figure 3), but none of them showed any clinical manifestations so far. The five carriers are as follows: Individual II-2 (the sister of the proband), a 57-year-old female; Individual III-4 (the son of the proband), a 28-year-old male; Individual III-5 (daughter of the proband), a 30-year-old female; Individual IV-1, a 6-year-old boy and Individual IV-2, a 3-year-old boy.

**Table 1:** Patient’s clinical characteristics according to the scale for the assessment and rating of ataxia (SARA)

Characteristics	Scores
Total motor score (range, 0–40)	18
Gait (range, 0–8)	3
Stance (range, 0–6)	3
Sitting (range, 0–4)	2
Speech disturbance (range, 0–6)	3
Finger chase (range, 0–4)	2
Nose-finger test (range, 0–4)	2
Fast alternating hand movements (range, 0–4)	2
Heel-shin slide (range, 0–4)	1

**Table 2:** Result of INAS count of the proband

Characteristics	Scores
Hyperreflexia	1
Areflexia	0
Extensor plantar	0
Spasticity	1
Paresis	0
Muscle atrophy	0
Fasciculations	0
Myoclonus	0
Rigidity	1
Chorea/dyskinesia	1
Dystonia	1
Resting tremor	1
Sensory symptoms	0
Urinary dysfunction	0
Cognitive dysfunction	1
Brainstem oculomotor signs	0

**Informed consent:** Informed consent has been obtained from all individuals included in this study.
**Ethical approval:** The research related to human use has been complied with all the relevant national regulations, institutional policies, and in accordance with the tenets of the Helsinki Declaration, and has been approved by the Biomedical Research Ethic Committee of Jinan Shizhong District People’s Hospital (approval number 20210429).

## 3 Discussion

SCAs are a group of neurodegenerative diseases with ataxia as the main clinical manifestation. Its core symptoms [18] are ataxia, nystagmus/retinal damage, and dysarthria. In addition to ataxia, SCAs also manifest as a variety of non-ataxia symptoms, which makes different subtypes show a high degree of heterogeneity. According to the genetic variation, SCAs are divided into three categories [19].

The first type is SCA caused by CAG repetitive amplification of the coding region [5]. Repeatedly amplified CAG is translated into polyglutamine (polyQ). PolyQ SCA includes SCA1, SCA 2, SCA3, SCA6, SCA7, SCA17, and DRPLA [20]. This category is the most common and widely studied. These subtypes show high regularity. First of all, in successive generations, when the number of repeated expansions of CAG gradually increases, the age of onset gradually decreases, and the phenotype gradually becomes serious. If the number of amplifications exceeds 100 times, multiple systemic lesions may appear in infancy [21]. Second, there is a threshold for the number of CAG amplifications, and it will be manifested if it exceeds this threshold. Generally, CAG amplification times within 35–40 can maintain a stable state [2]. Third, the variation in paternal origin leads to more serious consequences, especially in SCA7 [3]. But there are exceptions. For example, the number of amplifications of SCA8 tends to expand in maternal inheritance, but shrinks in paternal inheritance. The toxicity of the amplified polyQ protein is clearly present. Inhibiting the expression of the mutant protein in transgenic mice can alleviate the phenotype of SCA7 [1]. Toxicity mainly includes regulation of transcription levels, abnormal protein aggregation, degradation disorder, and other aspects [22,23]. (1) Yi-Cheng Lin believes that the toxicity of polyQ protein is first caused by abnormal transcription. The mechanism of action includes protein-protein or protein-DNA interaction, acetylation, phosphorylation, and RNA interference. It has been found that most ataxia proteins can bind to transcription factors, leading to changes in gene expression [4]. (2) The extremely long polyglutamine chain formed by translation will misfold and form aggregates. Aggregates can be located in the cytoplasm or nucleus, but the nucleus seems to be the place where the aggregates play a role [4]. Aggregates in the nucleus are considered to be a prerequisite for the negative effects of the mutant protein [24]. This seems to support the toxic effect of the mutant protein at the transcriptional level from the side [19]. CACNA1A, the C-terminal fragment of SCA6 protein, must enter the nucleus to induce disease [25]. (3) The degradation of abnormal proteins depends on two pathways: the ubiquitin-proteasome system (UPS) and autophagy [26]. UPS labeled the abnormal proteins first, and then degraded them. Mutant proteins may not affect the labeling of misfolded proteins by ubiquitin, but affect the degradation process [27], and even destroy the structure in UPS [28]. Autophagy is another way to degrade mutant proteins and reduce toxicity. Inducing autophagy and reducing toxicity through drugs can be used as a strategy to treat some SCAs [22]. The autophagy pathway through the phagosome-lysosome system plays a key role in the elimination of toxic proteins [23]. However, studies have shown that the autophagy in SCA3 is also inhibited. At the same time, autophagy may also damage normal neurons. Both these pathways are inhibited, which causes the abnormal protein to not be degraded, but to further aggregate, thereby aggravating the toxic effect.

The second type is the repetitive amplification of non-coding regions, including SCA8, 10, 12, 31, and 36 [1]. SCA8, which may be caused by repetitive amplification of non-coding CTG, is also classified as polyglutamine SCAs [29]. SCA8, SCA10, and SCA12 are caused by repeat expansion of non-coding CTG, ATTCT, and CAG, respectively [30,31], although the pathogenicity of SCA8 mutations is still controversial [32].

The third type is classic mutations, including point mutations of SCA13 and SCA27, deletion of SCA15, and frameshift mutation of SCA11 [1]. This type of SCAs may be the result of a combination of multiple mechanisms. Maintaining calcium homeostasis is almost the basis of all biological activities in cells. Alterations of calcium homeostasis in neurons can cause some SCA. Inositol 1,4,5-trisphosphate (IP3) receptors (IP3Rs) play a key role in regulating intracellular calcium concentration [33]. IP3R1 type (IP3R1) is widely distributed in Purkinje cells of the cerebellum. SCA15 and SCA29 lead to mutations in the ITPR1 gene encoding IP3R1 due to deletion or missense mutations, which in turn leads to disorders of calcium homeostasis [33].

With the widespread application of next-generation sequencing, this type of SCAs has been continuously discovered. Nibbeling et al. discovered FAT2 [34], Watson et al. studied GRM1 [35], and Tsoi H and Yu ACS reported the CCDC88C gene.

The proband in this report belongs to this type of classic mutation. Point mutant gene CCDC88C can encode coiled-coil domain containing protein 88 C, which activates the c-Jun N-terminal kinase (JNK) pathway and induces apoptosis by regulating the hyperphosphorylation of the JNK pathway. The clinical manifestations are mainly spastic ataxia. There is no specific age of onset. Both the proband II-3 and his sister II-2 have a heterozygous mutation in this gene, so it can be inferred that this mutation originated from their father or mother. The proband II-3 passed this mutant gene to his son III-4 and his daughter III-5. II-2 passed the mutant gene to her son III-3. Strangely, her daughter III-2 did not carry the mutant gene, but the sons of III-2, that is, both IV-1 and IV-2 carried heterogeneous variation. Is it because the boys’ father III-1 carries the same mutation, or do the two boys have the same point mutation as II-3? The probability of carrying the mutant gene in normal population was 0.000097. Therefore, generally speaking, the probability of these two conjectures is very small.

In clinical diagnosis of SCAs, in addition to the continuous development of genetic testing methods, related scales are also indispensable. SARA is used to semi-quantitatively assess the severity of a patient’s ataxia. The scale has a total score of 40 points. The higher the score, the more serious the ataxia [15]. Inventory of Non-Ataxia Symptoms (INAS) is commonly used to assess patients’ non-ataxia symptoms.

From the perspective of imaging, SCAs can be divided into three categories: simple cerebellar atrophy, olivopontocerebellar atrophy, and whole brain atrophy [12]. The degree of cerebellar atrophy matches the severity of ataxia [36]. Theoretically, the severity of cerebellar atrophy can be predicted through the SARA score. Although different subtypes tend to have different locations of atrophy, in general, cerebellar atrophy is the initial stage of intracranial lesions, gradually developing to structures outside the cerebellum, and eventually involving the whole brain.

There are several defects in this study. First of all, because of doubts about the results of individuals IV-1 and IV-2, we wanted to retest, but their mother III-2 refused. Probably because of the high cost and conservative thinking, the people in this pedigree did not actively cooperate with the examination. Second, due to economic and social reasons, the proband refused to further improve cognition and invasive examination. Third, the observation time is shorter. Although individuals II-2, III-4, III-5, IV-1, and IV-2 are heterozygous carriers, they did not show any discomfort. This may be because the age of onset has not yet been reached. Members of the Hong Kong family reported by Tsoi had a concealed onset after the age of 40, while the Polish case reported by Leńska-Mieciek had an onset after the age of 33. The age of several carriers (II-3, III-4, III-5, IV-1, and IV-2) is less than 30 years old. Therefore, we suspect that the five carriers in the case have not reached the age of onset. This requires long-term follow-up and observation by doctors.
