# Erratum to “Spinocerebellar ataxia type 40: A case report and literature review”

**DOI:** 10.1515/tnsci-2022-0216

**Published:** 2022-04-01

**Authors:** Fengyue Han, Dan Su, Chuanqiang Qu

**Affiliations:** Department of Neurology, Shandong Provincial Hospital Affiliated to Shandong University, Jinan, Shandong, 250100, China; Department of Neurology, Jinan Shizhong District People’s Hospital, Jinan, Shandong, 250100, China

In the published article “Han F, Su D, Qu C. Spinocerebellar ataxia type 40: A case report and literature review. Transl Neurosci. 12(1); 379–84. doi: 10.1515/tnsci-2020-0190,” the affiliations of the first author and corresponding author are marked incorrectly. The authors would like to correct the affiliation and apologize for any inconvenience that it may have caused. The affiliated hospital of Fengyue Han and Chuanqiang Qu should be the Department of Neurology, Shandong Provincial Hospital Affiliated to Shandong University.

